# Membrane Technological Pathways and Inherent Structure of Bacterial Cellulose Composites for Drug Delivery

**DOI:** 10.3390/bioengineering9010003

**Published:** 2021-12-22

**Authors:** Alfred Mensah, Yajun Chen, Narh Christopher, Qufu Wei

**Affiliations:** Key Laboratory of Eco-Textiles, Ministry of Education, Jiangnan University, Wuxi 214122, China; 7190707903@stu.jiangnan.edu.cn (A.M.); m18861824955@163.com (Y.C.); doll6000000@yahoo.com (N.C.)

**Keywords:** bacterial cellulose, drug delivery systems, membrane technology, critical aspects, modification pathways

## Abstract

This report summarizes efforts undertaken in the area of drug delivery, with a look at further efforts made in the area of bacterial cellulose (BC) biomedical applications in general. There are many current methodologies (past and present) for the creation of BC membrane composites custom-engineered with drug delivery functionality, with brief consideration for very close applications within the broader category of biomedicine. The most emphasis was placed on the crucial aspects that open the door to the possibility of drug delivery or the potential for use as drug carriers. Additionally, consideration has been given to laboratory explorations as well as already established BC-drug delivery systems (DDS) that are either on the market commercially or have been patented in anticipation of future commercialization. The cellulose producing strains, current synthesis and growth pathways, critical aspects and intrinsic morphological features of BC were given maximum consideration, among other crucial aspects of BC DDS.

## 1. Introduction

Researchers around the world are increasingly thinking smaller and smarter to solve some of the biggest problems in medicine with precise technologies, helping to open up new possibilities for nanomedicine and membrane technologies. Some of the most promising research work in nanomedicine is driven by a focus on nanomembranes which goes deeper into the engineering of functional systems at the molecular and atomic level [1,2,3], combining elements of material physics and molecular chemistry to derive unique properties that occur at the nanoscale level [4,5].

Academic articles, as well as registered patents in the past decade, show that cellulose nanomaterials have seen a great surge in laboratory and industrial exploration [6]. Nanocellulose is a unique cellulose variation, often consisting of long chains (1–4) linked β-D glucopyranosyl units organized into pyramidal microfibril structures [7]. They are otherwise referred to as “cellulose nanomaterials.” Their well-defined structural dimensions make them perfect for applications such as food packaging, flexible screens, thermo-reversible and tenable hydrogels, paper manufacturing, coating additives, optically clear films and lightweight ballistic protective materials, and automotive windows [8,9,10]. They are usually categorized as cellulose nanocrystals (CNCs), cellulose nanofibers (CNFs), and bacterial nanocellulose (BNC or BC). Nanocellulose displays unique qualities such as non-toxicity, biodegradability, and biocompatibility with no adverse impacts on health and the environment. This is why their drug delivery and biomedical applications have been fully explored. Custom-engineered systems that enhance epithelialization rates to aid in faster wound closure represent the future of regenerative medicine. These materials and their incorporated technology must regulate environmental conditions and increase cell adhesion, as well as proliferation, migration and differentiation [11]. Furthermore, they must possess important characteristics such as maintenance of a moist wound environment, gas exchange, thermal insulation, low tissue adherence among other qualities [12]. One practical example of this technology is the use of nanostructured biocellulose membranes (which is majorly represented by bacterial cellulose) to transport drugs to specific cells [13,14,15,16]. Aside from bacterial cellulose’s low toxicity and stable structure, they tend to be ideal containers for transporting drugs directly to the desired cells and have been found to demonstrate good in-vivo performance for wound healing, scaffolds, implants and drug delivery systems [17,18,19,20].

Introducing drugs into the human body may be accomplished by several anatomic routes. The choice of material, which further determines the most suitable administration route, is unquestionably important and leads to the ultimate goal of therapeutic success [21]. The drugs can be administrated directly to the target tissue or organ or can be delivered by systemic routes [22]. Drug delivery began long ago in the form of oral administration of solid pills or liquids, or sometimes injectables [23]. Long-standing problems with the initial administration methods led to new approaches and strategies developed to control several parameters considered essential for enhanced treatment performance like precise delivery, the rate, and time duration of delivery [24]. This marked the beginning of the now-called drug delivery systems (DDS). Natural and synthetic polymers have been studied and used in the preparation of DDS depending on their special features as well as their minimal or possibly no side effects in the course of usage or after usage. Koo and co. are of the opinion that the research community’s final focus is on developing controlled drug delivery systems that can be orally administered, be less expensive and less painful for the patient whilst being extremely effective [25].

Membranes have found diverse deployment in a vast array of industries and healthcare domains for years, aiding in solving complex problems [26,27,28,29,30,31,32,33]. In so much so that the world of medicine would have suffered a great deal in delivering custom-engineered solutions and precise target deliveries had membranes not been heavily researched, adapted, adopted and improved. Medicines, devices, procedures, and even organizational systems contribute to expanding life expectancy and improvements in quality of life [34,35]. Prescription drug treatment is forecasted to be sky-high at USD 1.2 trillion in market size by 2024, with the membrane-driven sector (medical membranes) globally valued at USD 2.73 billion in 2019 with more upscale research conducted on these materials. Medical membranes are used in a variety of applications, including drug distribution, bioseparation, tissue regeneration, and artificial organs or implants. [36]. Generally, there are natural membranes, otherwise referred to as biological membranes, and synthetic membranes. A membrane can be said to be a selective barrier that allows selected units or substances to pass through but stops others in principle. The substances referred to here could be ions, molecules, or other units of matter. They may occur naturally in nature or be derived by synthetic processes. Biological membranes include cell membranes (outer coverings of cells or organelles that allow passage of certain constituents) [37,38]; nuclear membranes, which cover a cell nucleus [39]; and tissue membranes [40,41], such as mucosae and serosae. Synthetic membranes are made by humans for use in laboratories and industry (such as chemical plants) [42]. Polysulfone, polyether sulfone, polyvinylidene fluoride (hydrophobic polyvinylidene fluoride, hydrophilic polyvinylidene fluoride), polytetrafluoroethylene, polypropylene, modified acrylics, and others make up the global medical membrane market. The most common material for medical membranes is polyvinylidene fluoride (PVDF).

An emerging class of membranes is nanostructured membranes, which have been used to fabricate hydrophobic membranes developed from polysaccharide and functionalized multiwalled carbon nanotubes (MWCNT), and which were applied for transdermal delivery of diclofenac sodium, doxorubicin, ifosfamide anti-cancer drug and a number of drug models [43,44]. Single-walled carbon nanotubes (SWCNTs) were used as a carrier to improve the anti-NNV activity of an immunomodulatory antiviral drug, isoprinosine, an anticancer agent, 5-fluorouracil, and a host of many drugs [45,46,47,48,49,50,51]. Carbon nanotubes (CNTs) have been successful as nanocarriers because they exhibit outstanding intrinsic physical and chemical properties which have seen them being extensively explored for versatile applications in recent years [52]. Single-walled and multiwalled nanotubes have proven to be good for desired drug delivery systems for gene transfer, treatment of cancer, transdermal, and DNA applications. Parameters such as structure, surface charge, agglomeration state, size distribution, surface chemistry, and surface area, as well as the purity of the samples, have a considerable role in the reactivity of CNTs [53]. Metal-organic framework (MOF) membranes, which are also a novel hybrid porous material composited by metal ions and organic linkers, have drawn increasing attention and have become a promising material in the biomedical field, obviously due to their unique properties such as large pore volume, high surface area, tunable pore size, versatile functionality and high drug loading efficiency [54]. Many drug models have successfully been incorporated into MOFs [55,56,57,58,59,60,61,62,63]. Several of them are MOFs for biomedical applications [64,65,66].

As their porosity comes from their peculiar extremely rigid backbone structure, polymers of intrinsic microporosity (PIMs) have been discovered to be a promising class of polymers for membrane separations and drug delivery [31,67,68,69]. Most of the preceding remedies have produced remarkable industrial and laboratory solutions. However, experts seem to assume that they might face varying degrees of environmental concerns.

There is a growing interest in developing natural polymer membranes based on nanocellulose, especially those based on cellulose nanofibers and bacterial cellulose [48,70,71,72,73]. Biological membranes, or biomembranes, are enclosing or separating membranes that function as a selectively permeable barrier within living things. A phospholipid bilayer consisting of embedded, integrated and peripheral proteins involved in the communication and transport of chemical products and ions is used to construct the cell membranes [74]. Bacterial cellulose (BC), which is an example of biomembranes, is a pure form of cellulose that can be synthesized by microorganisms, such as *Acetobacter xylinum* and *Gluconacetobacter hansenii* bacteria associated with *Saccharomyces cerevisiae,* under static or dynamic cultures [75,76]. Unlike cellulose nanofibers from other sources like wood pulp and agricultural residues, cellulose produced by an *acetobacter* strain is pure without other contaminating polysaccharides, and its process of isolation and purification is comparatively simple. They do not require intensive chemical processes coupled with not discharging harmful effluent into the environment like others. Excreted microfibrils from each synthetic site combine deep within the medium in which they are manufactured to form a long cellulose ribbon. The ribbons construct a floating pellicle, which enables non-motile aerobic bacteria to expand at a higher oxygen tension on the surface of the growth medium, which is then collected for subsequent treatment and use. BC has been used in a wide variety of applied research endeavors, including electronics, paper materials, acoustics, and biomedical devices, due to their unusual nanostructure, high purity, hydrophilicity, structure-forming ability, chirality, and biocompatibility properties. This has led to their becoming a natural candidate for various medical and drug delivery applications. Thus, this review elucidates the applications and streamlined modifications of BC membranes and other forms for drug delivery up-to-date. Further effort is invested in looking into their special innate structural features and how they contribute to effective drug delivery, which informs the choice of a specific administration route.

## 2. Bacterial Cellulose

BC is a polymeric nanostructured membrane used for diverse biomedical functions. Its 3-dimensional hierarchical non-woven network structure, combined with the fact that it is a naturally biosynthesized polymer discovered with exceptional chemical purity (free of lignin and hemicellulose), makes it an even more appealing option among many available biomaterials [77]. The additional cost and production steps required to purify plant-based cellulose limit its utilization in biomedical applications [78]. There has been such a sharp increase in the volume of scientific publications and citations reporting on BC for biomedical applications since 2000 [79]. 

A.J. Brown discovered BC in 1886, having initially observed it as “a jelly-like translucent mass on the surface of the culture fluid until it eventually formed a gelatinous membrane.” BC is a gram-negative rod-shaped aerobic bacterium that has been extensively studied due to its ease of fabrication, biocompatibility, high yield strength, and water retention properties, amongst many outstanding properties exhibited by BC [80].

**Figure 1:**
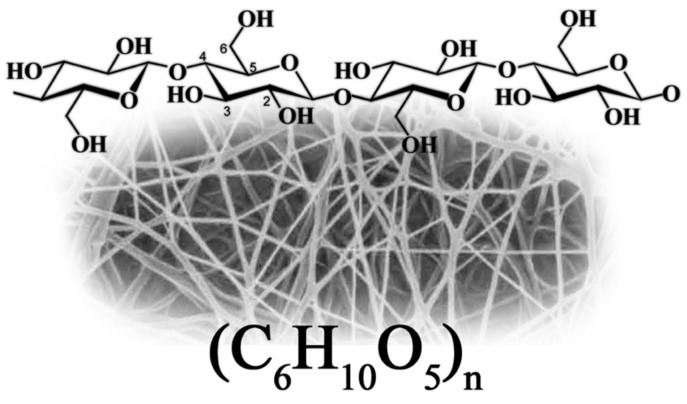
Chemical structure of Bacterial cellulose.

With an abundance of hydroxyl groups (OH) within its chemical structure as could be seen from Figure 1., BC serves as an enabling environment for the absorption and incorporation of other hydrophilic substances and nanoparticles [81]. There are growth contributors for cellulose from bacteria to be possible. The major one is the culture medium. The chosen bacterial strain is also known to affect the quantity and quality of BC synthesis, mainly based on genetics. It has been established that the presence of a specific operon encoding four proteins within *G. xylinus* leads to prolific BC synthesis [82].

Bielecki and co. add to the growth factors mentioned above that static incubation rather than agitation and aeration leads to better BC production in some specific strains [83]. Recently, as researchers have sought to bring BC to industry, advanced bioreactor-based production technologies have proven to be the way to go about it. Bioreactors, apart from increasing industrial-level productivity, ensure suitable control of the flow of the culturing media and aeration [8]. It is important to point out that it is still at the early stages of introduction to BC’s industrial production and so still requires numerous study efforts to optimize its usage in industry. 

Table 3, according to Lustri et al. shows that *Gluconacetobacter* species are prolific in production, fast in production, and establishes the notion that BC grows better and faster under static conditions [84]. Cultivating BC in an aseptic environment is highly recommended for its high functional and structural purity, especially if it is intended for medical applications [85]. 

### 2.1. Bacterial Cellulose Medical Applications and Commercial Usage

Cellulose from bacteria has been greatly used in the medical field, helping advance development in the field due to its valuable properties. BC is nontoxic, has excellent tensile strength, is porous, and has a microfibrillar structure. With a high aspect ratio of fibrils to give BC with a large surface area, the hydroxyl groups in the cellulose chains are closely linked to water molecules, thereby offering a high water retention capacity with water molecules attached to the hydroxyl groups within the cellulose chains [86]. Furthermore, as BC has an abundance of reactive groups, it may serve as a substrate for functions that may be either in situ or ex situ to fulfill different needs [78]. Many hydrogels, hydrocolloids, and bio or artificial membranes help in wound care because they provide the hydration required for effective tissue regeneration. A modern wound dressing should be non-toxic, nonpyrogenic, and biocompatible while also being model in order to provide a shield against infection, control dehydration, reduce pain during therapy, establish a moist environment in the wound, enable the entry or passage of drugs into the wound, absorb secretions during the proliferative reaction, and display good tensile properties, elasticity, and conformability. Bacterial cellulose exhibits almost all the properties mentioned above [85]. BC is both an effective barrier to outside infestation and a way to deliver antibiotics or other medications into the wound. It is capable of meeting the demanding specifications of contemporary wound dressing materials. Several companies have had success with utilizing BC in clinical therapies and implants. Biofill, a company in Brazil, has investigated and commercially produced two unique products called Bioprocess and Gengiflex, capitalizing on BC’s unique properties for wound healing. Chawla and co. report that a US company named Xylos Corp. produced Prima CelTM, a BC-based product for clinical ulcer and wound healing [87]. Many more pharmaceutical enterprises have had commercial-scale successes with BC as the main material or as a component, which has been shown in Table 1.

Because of their structure, hydrogels are especially well-suited for tissue engineering and medication delivery. BC that has been cleansed or purified may attain endotoxin levels that are as low as 20 endotoxin units per device, and the FDA has previously cleared it for implantable devices [88]. Mustafa and his fellows emphasize that BC is a key constituent in various FDA-approved wound dressings [20]. Over the last few years, Charreau and colleagues have reported on a number of patents (see Table 2) that have been filed for medication delivery systems that use BC [6]. A brand-new PVA–to be consistent with the heart valve’s physical features has been used in the medical field. the PVA-BC nanocomposite material has the same structural integrity as the porcine heart valve [72,89]. BC has been the subject of several studies and experiments, and might thus be a good candidate for creating synthetic blood vessels [90,91,92,93]. BC was investigated and explored as a contact lens for ophthalmic utilization [15,94]. A novel tissue-engineered cornea has also been developed from BC and polyvinyl alcohol (BC/PVA) hydrogel composites and experimented on for the reconstruction of an artificial cornea by Han and colleagues [95] and other studies on pure BC and BC composites for cornea regeneration [96,97].

Bacterial cellulose was used to treat epithelial tissues in studies conducted with lab colleagues [98]. Bacterial cellulose scaffolds were produced with varied oxidation degrees (O.D.s) for use in peripheral nerve healing, all with the help of sodium periodate ([99], as creatively reimagined, reconceptualized, [18,19,100,101,102,103]. A novel dural material, developed from bacterial cellulose (BC), was investigated in a rabbit model with dural defects for effectiveness and safety [104,105].

### 2.2. Bacterial Cellulose for Drug Delivery

**Figure 2:**
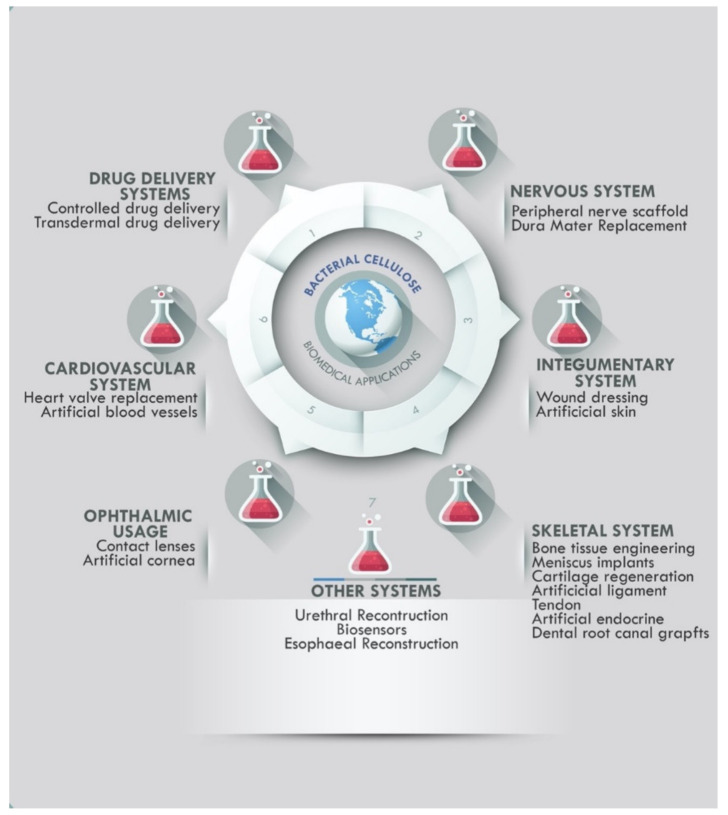
BC membrane technologies for drug delivery systems.

The complex yet fascinating process of delivering medications in a safe and efficient manner to ensure improved drug accessibility at the specified location with minimum adverse effects has been a frustrating but rewarding topic of biomedical study [17]. There is no unilaterally accepted definition for drug delivery systems, but attempts to define them hinge on the following principles: a single or multiple drug compound, the custom technology that carries the drug and delivers them into the body (medical device or dosage form) or target area and the drug-release mechanism. Drug delivery using nanotechnology or nanomembranes is a novel and promising strategy in therapeutic medicine. The properties of the nano-based carrier that aid in the efficiency of drug delivery systems include encapsulation capabilities, hydrophobicity, hydrophilicity, biocompatibility, bioavailability, biosafety, and therapeutic efficiencies such as pharmacokinetics, cellular uptake, controlled distribution or release, excretion, toxicity, and clearance. As disease and ailment tend to be complex, more dynamic, robust, adaptive and efficient systems have had to be engineered [1,14]. Development of these systems have comprised the study, the designing, the creation, synthesis, effective and thorough characterization, manipulation and application of materials, apparatus and systems by controlling the structure of materials at the nanoscale [80,86]. What used to be exploration at the micro level has in recent times morphed to atomic or molecular level exploitation. As these materials are probed and manipulated at nanoscale, the material properties change with a good level of focus on not generating side effects along the way. Figure 2 below shows drug delivery systems as one of the major branches of BC’s biomedical applications.

Some recent BC and BC composites for specific drug delivery or with potential for drug delivery are discussed in this chapter. Through modes such as cross-linking reactions, grafting, reactivity via hydroxyl sites, mineralization, and many other mechanisms, BC has seen utilization after being deemed drug-holdable or intrinsically therapeutic. 

**Figure 3:**
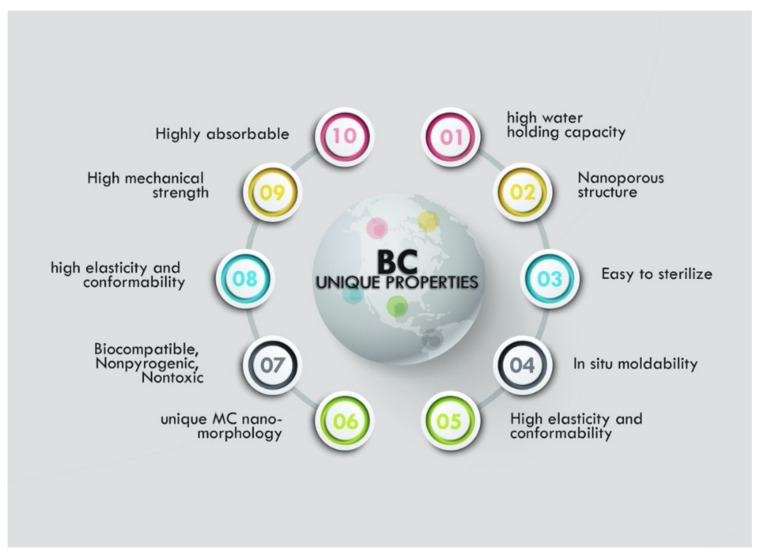
The unique properties of bacterial cellulose.

These modification modes augment the unique features of BC, rendering it with intrinsic features and properties such as high water holding capacity, a much slower water release rate, high crystallinity, great tensile properties, ultrafine fiber network, better thermal and mechanical properties, hydrophilicity, polyfunctionality, transparency, nontoxicity, and moldability into three-dimensional structures. These features and properties make BC a much-preferred choice currently over cellulose from plants as a carrier system for drug delivery. Figure 3 shows the unique properties of bacterial cellulose.

Based on the solution impregnation procedure, BC membranes with laser-sensitized magnetic nanoparticles (LMNs) were used to improve the efficacy of a breast cancer medication targeted therapy. An in situ transdermal administration device that synchronizes hematoporphyrin monomethyl ether (HMME) and doxorubicin (DOX) delivery to a breast tumor was successful [86]. Doxorubicin, a cytotoxic medication, was incorporated into BC/NLC hydrogel matrices together with bacterial cellulose (BC) and nanostructural lipid carriers (NLCs). Cell internalization and cytotoxicity of NLCs loaded with cationic Dox (NLCs-H) or neutral Dox (NLCs-N) were completely defined, as were their in vitro cellular and cytotoxic effectiveness, on MDA-MB-231 cells. They were successfully tested in vivo on an orthotopic breast cancer mouse model [14]. Benzalkonium chloride, an antibacterial agent, was added to a freeze-dried BC film, which then was submerged in a solution of the cationic surfactant benzalkonium chloride. The drug-loading potential of the BC dry film was determined to be around 0.116mg/cm2 when submerged in 0.102% benzalkonium chloride solution, intended for drug delivery. Another important aspect is the high water absorption ability of the resultant composite materials, which is essential for wound dressings [106].

A novel composite material consisting of calcium-deficient hydroxyapatite (CdHAP) biomimetically deposited in a bacterial cellulose hydrogel was synthesized and characterized with the potential to be used as a template for biomimetic apatite formation (as an orthopedic biomaterial) [26]. Images of the composites confirmed uniform ~1 um spherical CdHAP particles comprised of nanosized crystallites with a lamellar morphology formed within the cellulose matrix [107].

In a study [108] which investigated a novel strategy of adopting a simultaneous grafting/vinyl polymerization process to chemically anchor a quaternary ammonium salt (R-N(CH3)+) with a special vinyl group (2-methacryloyloxyethyl trimethylammonium chloride, METAC) onto the BC, an excellent water absorbability and a 99% antibacterial activity against *Escherichia coli* and *Staphylococcus aureus* were achieved. An excellent in-vivo antibacterial efficacy meant the composite could potentially be utilized as bio-safe, adaptive antibacterial surface for various biomedical applications. Amoxicillin (AM) loaded BC sponges were examined for wound dressings. Results revealed the AM grafted BC sponges to be promising wound dressings with excellent antibacterial property and non-toxicity; effective against *E. coli, C. albicans* and *S. aureus*, and non-toxic to HEK293 cells [109]. 

Charreau et al. [6] captured a slew of patents covering BC and BC composites for drug delivery and a variety of other biomedical functions; medicinal pads for topical application engineered via BC impregnated with a physiologically acceptable liquid; BC for biomolecule immobilization; oral BC plasters for stomatitis treatment; hollow BC useful in novel artificial blood vessels and other medical materials; and high absorption composites such as nappies and sanitary products for children. BC-transparent polymeric composites for use as osseous tissue support material; BC blood vessel prosthesis; artificial skin biomedical material made of BC membrane and poly(beta-hydroxyethyl methacrylic acid); implants for culturing cells; viable cell sheets and implants for cornea repair, cartilage repair, connective tissue repair, and ligament repair; bone cement to fix bones to prevent infectious diseases; and BC powder carriers as medicinal agents are also examples [6].

### 2.3. Critical Aspects Vital for BC-DDS and Biomedical Applications

**Figure 4:**
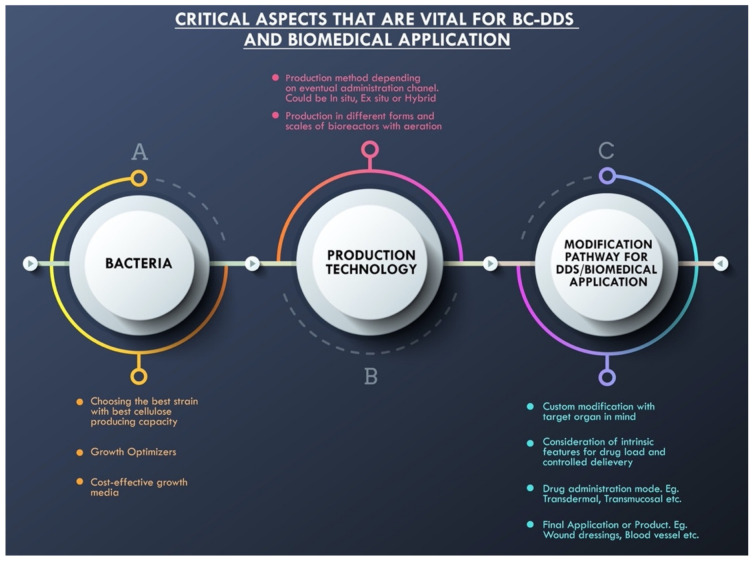
Illustration of the critical aspects vital for BC-DDS and biomedical applications.

This encompasses the vital factors for a successful drug delivery system using BC composites. Figure 4 illustrates these factors for concise viewing. From selecting the specific bacterial strain to the choice of production method or technology, and finally to inculcating the intended modification to the substrate, which determines the unique intrinsic properties critical for the drug to be delivered to target organs, every step requires critical planning and choice of technology for guaranteed success. Choosing BC as a substrate or component of a drug carrier first begins with the choice of bacteria species and/or strain. This is because the efficiency, productivity, and effectiveness of the final drug delivery system can be determined by the strain and its mechanisms of biosynthesis, ultimately leading to the eventual binding effect and strategy of drug holding for the entire delivery process being achieved successfully. The type of strain or genus, as well as the accompanying optimum growth factors and biosynthesis, have been adequately discussed in the ensuing sections. It is safe to mention that a more cost-effective growth medium is highly recommended.

Moving on from the choice of bacteria strain, drug delivery systems require precise enhanced characteristics for success. In doing so, careful consideration is essential when choosing the production technological path. Depending on the final mode of drug administration, adaptability and swift transformation from either hydrophobicity to hydrophilicity or vice versa, the engineering path has to be carefully designed. The contact angle in certain key moments of the drug delivery holding and release sequence is also determined by the technology used in fabrication. For these reasons, and many other intrinsic properties that BC-DDS should demonstrate in split-second vital moments, it is important to choose the right production strategy or method during the engineering stage. Current choices have been between either an in situ or ex situ fabrication path. A third path, a hybrid approach which essentially combines either both or selected stages of both is gradually gaining traction. Moreover, choosing between static, agitated, or bioreactors in the process of composition undeniably affects the eventual characteristics of the final system, and this, in turn, affects the carrier’s capability to hold the chosen drug, penetrate various media to the target organ or cell, and release the drug for the therapeutic effects to take place. This has also been amply discussed in subsequent sections.

The concluding aspect, which remains equally critical for BC-DDS, has to do with the factoring of the BC modification mode for delivery to be facilitated or enhanced. This, coupled with the technology method also determines the unique intrinsic morphological feature of the final system. They could take the form of a bead-like appearance, a sponge-like appearance, a transdermal or transmucosal unit, the 3D nanofibril network that is typical of bacterial cellulose, drug-impregnated lozenges, spherical pockets, or soft oral gels. These unique dispositions of the final system hold the key to the efficient, complication-free, and controlled delivery of drugs through the skin, or mucosal membrane, or nasal cavity, orally, through the central nervous system, or many other possible routes. Wound dressings tend to be more sponge-like or 3D networked in morphological appearance. Coated nanocrystals and cross-linked scaffolds are effective for controlled drug delivery in vivo and in vitro, and all the capabilities are determined by the chosen mode of modification. Modifications can be done via cross-linking reactions, grafting, mineralization, etherification, or esterification, among other modes of modifications. All these critical factors have been thoroughly discussed in the following pages.

#### 2.3.1. Bacteria

##### Bacteria Strains and Growth Factors (Biosynthetic Pathways) 

Cellulose-producing microbial strains (BC) utilize nitrogen and carbon sources for successful production. Normally, carbon sources are from glucose but can also be from other carbon sources such as sucrose, fructose, mannitol, and molasses [75,110,111,112]. Over time, fruit juices and other sources like 5- or 6-carbon monosaccharides, oligosaccharides, glycerol, starch, alcohol and organic acids have successfully been explored for BC growth [113,114,115]. With an increase in initial glucose concentration, the yield of cellulose decreased compared to the glucose absorbed, and gluconic acid accumulated at a high initial glucose concentration [75]. The decrease in cellulose yield could be due to some glucose being metabolized to gluconic acid. The optimum pH for cellulose production is between 4.0 and 6.0. Any glucose can be metabolized to gluconic acid, resulting in a decrease in cellulose yield. The ideal pH range for cellulose output is 4.0 to 6.0. Depending on the physiological state of the culture, glucose is metabolized by the pentose-phosphate cycle or the Krebs cycle [81]. The oxygen supply controls cellulose production, which is unaffected by carbon source concentration [84].

Despite the comprehensive study of BC, there is no single system that ideally represents the study of cellulose biosynthesis. Some well-researched genera of bacteria (refer to Table 3) that have successfully synthesized cellulose include *Gluconacetobacter xylinus* (formerly known as *Acetobacter xylinum*), *Agrobacterium* [116], *Aerobacter* [117], *Achromobacter* [118], *Azotobacter*, *Rhizobium* [119], *Sarcina* [117], and *Salmonella* [87]. Most of the species named above are gram-negative bacterial, with others being *Pseudomonas* and *Alcaligenes* [77]. The gram-negative bacterium *Gluconacetobacter xylinus* is one of the most researched genera; it secretes large quantities of cellulose as microfibrils from a row of synthetic sites along the longitudinal axis of the cell [120,121].

**Figure 5:**
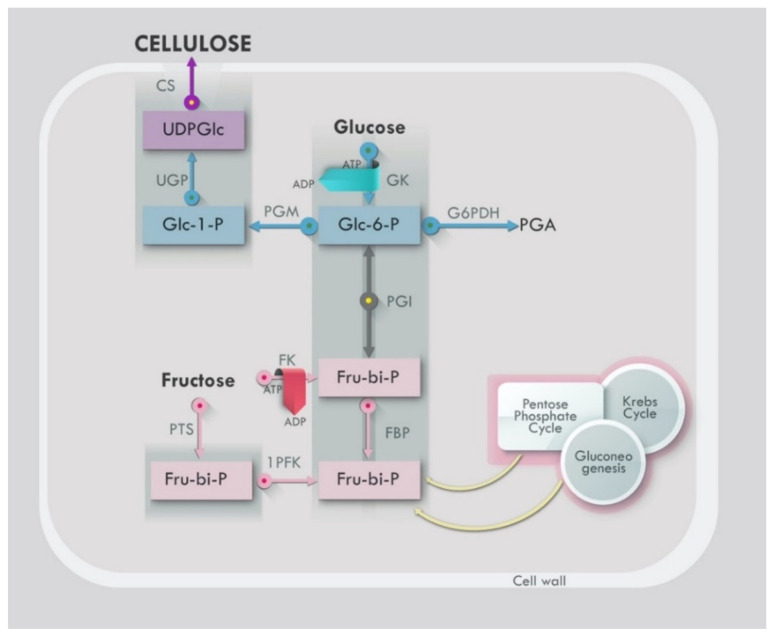
General biosynthesis path of bacterial cellulose.

This schematic, Figure 5, represents the general synthesis pathway of BC. There are four critical enzymatic conversion phases involved in the production of BC [77,81]. In the first phase, glucose-6-phosphate is glucokinase-phosphorylated, and glucose is produced (Glc-6-P). Phosphoglucomutase isomerizes Glc-6-P to glucose-1-phosphate in the second phase (Glc-1-P). The third step involves UDP-Glc-Phosphorylase (UGPase) which synthesizes uridine diphosphoglucose (UDP-Glc). Cellulose synthase then uses this to manufacture cellulose. The bacterial-derived cellulose synthase protein complex, which consists of two main subunits, performs the cellulose synthase enzymatic process. c-di-GMP binds to BcsA (an inner wall-anchored periplasmic protein) at the active site of cellulose synthase, and binds to BcsB (an inner wall-anchored periplasmic protein) outside the active site. BcsA’s catalytic activity is reliant on BcsB. Cellulose synthase is the last stage of the process, which ends with the synthesis of cellulose [77]. Complex formed by these two enzymes is required for cellulose production. Subunits of two additional types also exist. The transmembrane pore BcsC is responsible for providing the microfibrils for cellulose crystallization, and the periplasmic soluble protein BcsD is essential in creating those microfibrils [29]. The manufacture of cellulose may be carried out using purified BcsA and BcsB proteins, while mutations in BcsC and BcsD lower the yield of produced cellulose. UDP-Glc is a direct cellulose intermediate that is found in many organisms. UGPase, like UDP-Glc, is involved in cellulose synthesis and yet is 100 times more active in cellulose-producing bacteria than in noncellulose-producing bacteria. Cyclic diguanylic acid (c-di-GMP), an allosteric activator of cellulose synthase, is also involved in the development of BC [121]. Cellulose synthase remains inactive or has low enzyme activity in the absence of c-di-GMP [137].

Recently, there has been an abundance of visualizations of the cellulose synthesis pathways and membrane translocation, providing significant knowledge about the complete process (in situ). After evaluating many different studies, we came to the conclusion that we would exhibit many interesting versions of DDS with distinctive properties that are dependent on the species of bacteria selected and the morphology they form that aids in the administration of drugs.

**Figure 6:**
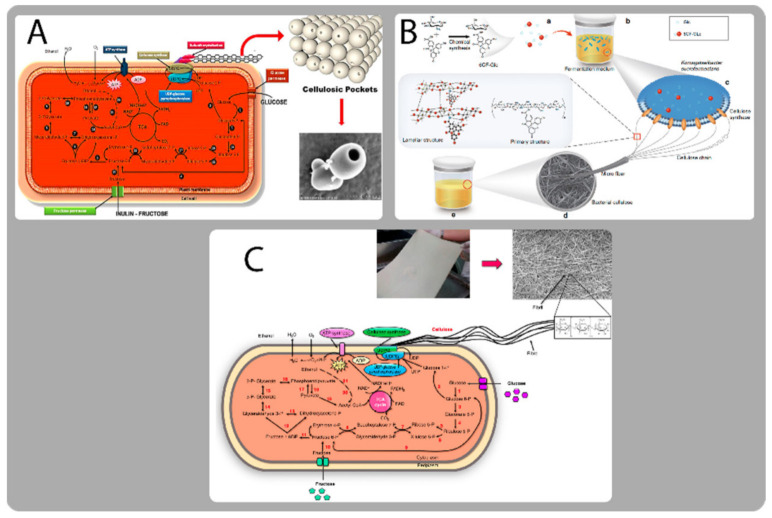
Visualization of various biosynthesis paths of bacterial cellulose. (**A**) Bacterial cellulosic (BC) pocket synthesis mechanism reported by Narh et. al. [138]. (**B**) Synthesis of 6CF-BC based on an in situ microbial fermentation method reported by Gao and co. [139]. (**C**) Pathways for the biosynthesis of BC by *K. xylinus* and the assembly of cellulose molecules into nanofibrils reported by Jacek et al. [140].

Narh and colleagues created bacterial cellulose pocket (BCP) carriers for medication delivery that remain stable over time (see Figure 6A). This ATCC 10,245 strain of *Gluconacetobacter xylinus* was used with inulin to serve as their fructose permeate. Averaged examination shows that the pockets may be used to carry nanomolecules, and hence they might possibly be used to provide medications and other chemicals [138]. They explain that the fructose units in the inulin compound are joined by a β(2→1) glycosidic bond, making it unusually flexible and capable of assuming a variety of structures, which in their study formed “pockets” that could potentially encapsulate drug models. In the end, empty bacterial cellulosic pockets (dimensions: between 1 to 3 um) with an entrance width of about 150 nm were successfully synthesized by introducing inulin to the fructose permeates of the *Gluconacetobacter xylinus* strain. 

Another article described a technique to synthesize pyrimidine ribonucleotides (a kind of pyrimidine nucleoside) that might be an alternative to genetic engineering. Figure 6B is a vivid illustration of the synthesis path in this study. A nonnatural characteristic fluorescence was introduced into a bioluminescent BC using the enzyme *Komagataeibacter sucrofermentans* [139]. In their unique innovative modification method, Glucose is functionally modified with 6-carboxyfluorescein (6CF) and used as a substrate to produce the functional BC by in situ fermentation with *K. sucrofermentans*. Although not directly related, adjusting the content of 6CF-modified glucose (6CF-Glc) in the culture media can alter the fluorescence intensity of functional BC. This provides an insightful blueprint for genetically designing BC drug delivery composites. Functionalization of BC has enormous promise for biomedical applications through biochemical modification.

In their article on the biosynthesis of cellulose nanofibrils by the thermophilic *Komagataeibacter xylinus* organism published in 2019 [140], the metabolic route in *Komagataeibacter xylinus* that produces cellulose nanofibrils is elucidated. This figure is really informative, especially in terms of showing all of the steps in the complex multi-step-controlled process shown in Figure 6C. In this figure, you can see that the production of b-1,4-glucan chains involves the actions of multiple individual enzymes and protein complexes of catalytic and regulatory proteins, and that these steps lead to the crystallization of cellulose, the production of glucose, and the conversion of glucose to cellulose via four enzymatic steps: phosphorylation of glucose by glucokinase to G6P; isomerization of G6P to G1P by PGM; conversion of G1P to UDP-glucose by UDP-glucose pyrophosphorylase; Bcs A, Bcs B, Bcs C, and Bcs D, the subunits that comprise Bcs, are all encoded by bcsAB, bcsC, and bcsD. This claim asserts that the key enzyme in cellulose production is UDP-glucose pyrophosphorylase, which does a hundred times more work in cellulose-producing bacteria than it does in non-producing bacteria [141].

##### Bacterial Cellulose Structure and Unique Properties

This kind of polysaccharide which is produced by bacterial cells, is formed of D-glucose rings connected by β(1-4) bonds, with five carbons and one oxygen in a ring of six atoms [142]. These microfibers are extruded in between the outer and cytoplasmic membranes, growing from 2 μm each minute to reach their full size [143]. A microfibril strand may be 1.5 nanometers broad, and have an intricate pattern that helps bacteria to guide their own self-assembly. The smaller microfibrils that have diameters ranging between 30–50 nm will develop into bundles [144].

BC ribbons have been reported to have different diameters by Bielecki and colleagues; 3 ± 4 thickness ×70 ± 80 nm width, 3.2 × 133 nm and 4.1 × 117 nm, proving that depending of conditions and nutrients bacteria cells may produce varied dimensions of ribbons [83]. As monomicroscopic ribbons of microbial cellulose are generated, they are maintained by substantial hydrogen bonding, which may range from 1 to 9 monomicroscopic ribbons in length. As a consequence of the presence of many hydroxyl groups, the production of characteristic insoluble cellulose polysaccharide chains of BC occurs. Intra-chain hydrogen bonds as well as inter-chain hydrogen bonds allow for the development of polysaccharide sheets composed of stacked sheets of cellulose that are mechanically connected to one another by weak van der Waals forces. Dispersion forces occur between the stacked heterocyclic monomer rings to strengthen the cellulose sheets [142].

The morphological structure of BC is strongly influenced by culture conditions [145]. Cellulose I and cellulose II have two major crystalline structures. They are also known as parallel and anti-parallel cellulose chains. A few different technologies are used, such as NMR, X-ray, and Raman spectroscopy. Mercerization or alkali treatment (also known as alkali ligation) yields a more thermodynamically stable structure when used on cellulose I. Cellulose I, broken down into its two constituent amorph structures, cellulose Ia and cellulose Ib, is further broken down into two even more amorphous forms: cellulose II and cellulose III. Cellulose Ia is a stable phase of cellulose with a two-chain monoclinic unit cell, whereas cellulose Ib is a meta-stable phase of cellulose I with a triclinic unit cell [142]. as much as half of all plants, are composed of this in static culture, *xylinum* has been seen to produce cellulose. Cellulose I have uniaxially-organized, parallel β-1,4-glucan chains, while cellulose II has randomly-organized β-1,4-glucan chains. Despite the above statement, cellulose II has much stronger thermodynamic stability [145]. 

Cellulose I is a prominent plant compound, which is synthesized by the majority of plants, especially *A. xylinum* in static culture. Cellulose I has uniaxially-organized, parallel -1,4-glucan chains, while cellulose II has randomly-organized β-1,4-glucan chains. Despite the above statement, cellulose II has much stronger thermodynamic stability [145].

As the overall cellulose composition swaps and alternates between the various amorph phases of cellulose, cellulose fibers are said to be extruded from the bacterium and aligned arbitrarily. Cellulose Ia has intra-molecular hydrogen bonding between O_3_-HO_5_ and inter-molecular hydrogen bonding between O_6_-HO_3_, while cellulose Ib can be said to have intramolecular and inter-molecular hydrogen bonding between O_6_H-O_2_ [146]. Cellulose Ib has been discovered to be more thermodynamically stable than cellulose Ia under a high-resolution solid-state carbon-13 nuclear magnetic resonance (13C NMR) because they are irreversibly formed from cellulose Ia and have doublets at C-1, C-4, and C-6. Cellulose Ia on the other hand has singlets, or paired electrons, at the C-1 and C-6 with a closely spaced doublet, or an unpaired electron, at C-4. 

Zyl and co. report that BC has the highest concentration of cellulose Ia polymorph at 70%. According to Ross et al., BC polymer structure is determined by the specific organism used, but the biosynthesis and principal mechanism of regulation within the synthesis medium remains similar for all strains [121].

#### 2.3.2. Production Technology

##### Preparation Methods and Strategies for BC DDS Membranes

BC is a highly biocompatible material that lacks appropriate functionalities to trigger initial cell attachment. Again, control over the porosity and their slow degradation have slowly been found to be further obstacles in the way of BC’s ever-growing versatility and usage in industries, including for drug delivery system. Chemical means (modification of chemical structure and functionalities) and physical means (change in porosity, crystallinity and fiber density) by way of applying adaptable ‘in situ’ and ‘ex situ’ strategies were the way-out years ago. In general, these two main strategies, in situ and ex situ were considered to be the main methods in producing BC composites for drug delivery. However, in the process time and researching for this review, we discovered a third option slowly making way into mainstream BC DDS composite preparations. We henceforth include it as a third alternative. It is a ‘tandem’ or ‘hybrid’ of the two initially utilized methods employed in engineering a unique composite for special applications in biomedicine. The hybrid approach makes a strong case for itself [147]; which is why we believe researchers should be looking at it more alongside the established approaches. As a result, in situ, ex situ, and a hybrid technique are the three basic strategies for creating BC DDS-based composites. Each strategy is used depending on the final utilization of the composite. 

In situ modifications entail usage of variations of culture media, carbon source and introducing other vital alternative materials to help engineer the desired properties right from the formative stages of the composites, while ex situ modifications are undertaken for chemical and physical treatment of already harvested BC. Under ex situ strategies, two possibilities are explored. One, is the modification done to the washed BC pellicle which may or may not be freeze-dried prior to the further modification. We refer to it here as ‘unprocessed pellicle’ approach under ex situ modification, hence, called Ex situ ‘unprocessed pellicle’ approach. The other, is modification via BC suspension or solution approach; by which the washed pellicle gets further processed into suspension or solution forms before a following modification is done. In this case, the pellicle may be ultrasonicated into powder or granular-like form before any further chemical introductions. A strong case is made for the division of the ex-situ strategy of modification of BC, which was both initially referred to as ex situ methods. We believe the distinction is necessary because, as shall be clearly distinguished later under this chapter, modified unprocessed pellicles (ex situ ‘unprocessed pellicle’ approach) composites have a unique morphology which is quite different when compared to ‘further processed’ pellicle (ex situ suspension/solution approach) composites. There are many distinct approaches used for the preparation of ex situ-prepared cellulose-based DDS such as desolvation, electrospraying [148], spray-drying [149], layer-by-layer self-assembly [150,151], supercritical fluid extraction [152,153], freeze-drying and microemulsion [154] among others; with each chosen method having its own pros and cons.

(a) In situ pathway

In situ preparation strategy is the most commonly used amongst the methods as we thoroughly searched online. It seems to be the shortest and most less-cumbersome approach. To say something is “in situ” originally is a Latin expression. It may imply “on site”, “targeted” or “in the local position” in medical terminology. In surgical situations as well as in cancer diagnosis and therapy, the word is used interchangeably. In biology, ‘in situ’ refers to a phenomenon that occurs exactly where it happens; in this specific case, in situ cultivation refers to the entire biological machination that takes place in the medium environment to produce the final pellicle. The time period for cultivating depends on the thickness of membrane desired and the particular bacteria strain used for the composite synthesis. This approach exhibits several advantages as it involves introducing modifiers into the culture medium to be interlocked within the BC matrix engendering a physico-chemical modification in the process of cellulose formation. The modifier materials become part of the fibrils which also enhances the BC by altering mainly the physical–mechanical properties of BC fibrils. A successful incorporation changes the end-functionality and properties of the BC. 

Ultimately, the choice of culture medium conditions and method of deriving cellulose produced by bacteria becomes crucial factors when considering the in situ pathway. BC production is authentically an in situ process and depending on the final utilization of the BC and properties required, explorers had to settle for either a static or agitated cultivation approach. However recently, industrial scale production became necessary due to BC’s growing acceptance in multiple fields as a result of it proving to be a versatile material, leading to a demand for a much more robust, ultra-productive setup. Bioreactors emerged as the solution; a third and more industrial level solution. The supramolecular structure of BC and its mechanical properties can be directly influenced by its production method [122]. Figure 3 depicts the whole range of unique features of bacterial cellulose as described in the literature we reviewed. Over the years, static cultivation approach has tended to be a standard method, resulting in highly homogeneous supramolecular BC structures. It is mostly chosen because it synthesizes high quality structures with good properties for end uses as they are harvest with a flat appearance. Static cultivation is the simplest amongst the three and has seen an overwhelming deployment in the engineering of drug delivery systems as evidenced on Table 4. Agitated cultivation approach has also been decently used in the field of drug delivery and other biomedical applications due to special beadlike features derived. It is known to typically produce cellulose rapidly than a static method. Simple fed-batch was introduced as new culture system as strategy to increase the BC productivity suitable for commercial applications [155], then bioreactor for a semi-continuous production came on the scene [156] after a modified airlift-type bubble column bioreactor had emerged earlier [157]. Bioreactors solidified the industrial scale production of BC although most researchers recommend further studies to optimize its usage in industry. Using bioreactor ensures suitable control of media flow and aeration which helps in proper growth of microorganisms or animal cells according to Sharma and co. [122]. A plethora of in situ BC-DDS have been reported in many lab-scale studies.

**Figure 7:**
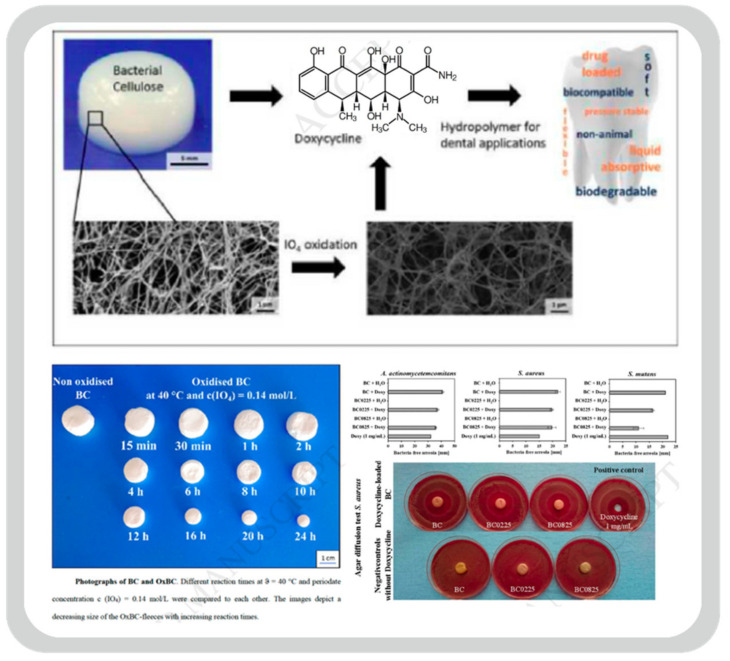
Illustration of a wound cover made from modified bacterial cellulose for dental therapies. Adapted with permission from [158].

A static cultivation approach was done by Weyell and co with *Komagataeibacter xylinus (K. xylinus)* strain DSM 14666 [158]. Using the Hestrin–Schramm culture medium (HSM) [75], this *K. xylinus* strain was inoculated for 14 days at 28 °C, loaded with a drug model Doxycycline for dental therapies after periodate-oxidation. Figure 7 shows the result [158].

**Figure 8:**
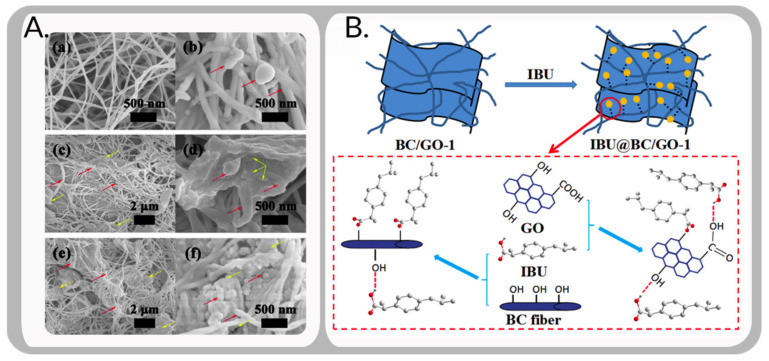
Illustration of bacterial cellulose/graphene oxide nanocomposite as a novel drug delivery system: (**A**) SEM images showing the unique morphological features with the 3D structure: (**a**), IBU@BC (**b**), IBU@BC/GO-1 (**c** and **d**), and IBU@BC/GO-2 (**e** and **f**) (red arrows indicate IBU and yellow arrows indicate GO).; (**B**) the mechanism of surface interactions between the BC/GO and IBU drug models. Adapted with permission from [159].

A team from a university in China incorporated an evenly distributed a graphene oxide (GO) layer into the 3D pore system of bacterial cellulose (BC) to make a new BC/GO nanocomposite drug carrier system with ibuprofen (IBU). In this striking figure (Figure 8), BC/GO nanocarriers were generated on-site for 10 days under 30 °C static conditions. Graphene acts as the modifier in the composite, while the bacterial cellulose side (BC) acts as the matrix material on the outside. The BC strain in the research was *Komagataeibacter xylinus X-2* [159]. The inclusion of GO as a useful intermediary improved the established IBU release behavior, further supporting the positive effect of GO in reducing pressure. In contrast to Weyell and co., the researchers disclosed a completely distinct morphology, with a nanosheet ball-like extension appearance shown by SEM images (at a nanoscale magnification).

Faria and co. used in-situ free radical polymerization of glycidyl methacrylate (BC) to prepare nanocomposites of poly (glycidyl methacrylate) (PGMA) and bacterial cellulose [160]. Following post-modification using acid-catalyzed hydrolysis, the hydrophobic PGMA component was rendered hydrophilic, resulting in greater hydrophilicity suitable for clinical therapies. Morphological scans revealed that the nanocomposites had irregular-shaped microsized forms similar to those captured by Luo et al. [159].

**Figure 9:**
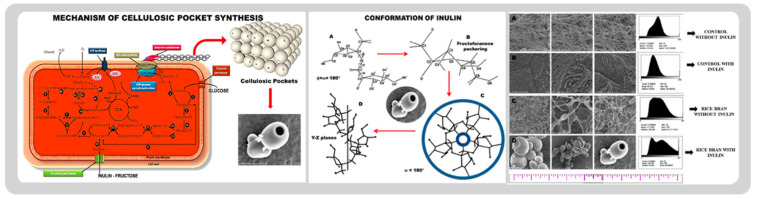
Presentation of bacterial cellulosic pocket synthesis mechanism and inulin conformation. Pocket dimensions and morphology are well illustrated: (**A**) is the inulin D-fructose molecule in all-trans conformation of ϕ = Ψ = ω = 180 while (**B**) is the ring puckering with C3 atom displacement. (**C**) is the proposed inulin conformation chain X-Y whereas (**D**) represents the Y-Z planes. Adapted with permission from [138].

Following Figure 9, Narh and co. made modifications to bacterial cellulose culture medium constituents by introducing inulin to create nanosized pockets (BCP). *Gluconacetobacter xylinus* (ATCC 10,245) bacteria produced hollow cellulose pockets which can be exploited for medicine storage. There were a variety of pocket diameters, ranging from 1 to 3 um, with an entry width of roughly 150 nm [138].

Researchers at Ciechańska’s laboratory researched a blend of bacterial cellulose and chitosan composite materials with the appropriate proportions of glucosamine and N-acetylglucosamine to be applied to the outside of the human body as an ideal wound, blister, and ulcer covering. The specific Bacterial strain (*Acetobacter xylinum* (*ŁOCK 0805*)) eventually demonstrated good wet tensile strength, excellent humidity control, lysozyme-mediated release of mono- and oligo-saccharides, and bacteriostatic activity against both Gram-positive and Gram-negative bacteria [161].

Romanov et al. prepared organic-inorganic composite materials with different nanotextures using three methods based on two nanosized and biocompatible compounds, cellulose *Gluconacetobacter xylinus* (CGX) and hydroxyapatite Ca_5_(PO_4_)_3_OH (HA) [162]. By varying the quantitative ratios of the components and the methods of incorporating HA into composites, a diverse range of materials for medical applications was developed.

Bacterial nanocellulose (BC) produced by the bacteria *Gluconacetobacter xylinus* is synthesized and impregnated in situ with iron oxide nanoparticles (IONP) (Fe3O4) to yield a magnetic bacterial nanocellulose (MBNC). The synthesis of MBNC is a precise and specifically designed multi-step process [163].

Other work developed with the modification method utilizing the in situ microbial fermentation method includes BC functionalized with magnetite and hydroxyapatite as nanoparticles for bone tissue engineering [164], BC/carboxymethylcelullose (BC/CMC) biocomposites developed as drug delivery systems [165], BC/GO pellets composite were prepared as drug carriers [166].

Despite its widespread use and various benefits, the in-situ alteration technique has a few significant limitations. First, incorporating antibacterial reinforcing materials toward BC strains can be difficult, as can the insolubility of different materials in culture media, high surface tension against hydrophobic materials, lack of structure regulation of BC nanofibers, and the introduction of particles with poor suspension stability into BC expanding media, among other issues.

A more detailed overview of the current main systems can be seen in Table 4 below.

(b) Ex situ ‘unprocessed pellicle’ pathway

Ex situ is the exact opposite of in situ and describes processes away from the natural location, in this context, the cultivation medium. A distinct difference between in situ and ex situ is that the experimental conditions are difficult to maintain in in situ methods, whereas with ex situ methods, experimenters have an increased level of control over the experimental conditions. They can be easily maintained and creatively manipulated. As stated earlier, ex situ modifications are mostly chemical (e.g., periodate oxidation, grafting or crosslinking reactions), which don’t necessarily require the pure (wholesome) pellicle to undergo further physical processing before modification, and as well as physical (physical absorption from solutions or particle suspensions, the homogenization or dissolving of BC, mixing with additive material), then modifications can be undertaken.

We here again emphasize that the two ex situ approaches should be distinguished, and we describe the Ex situ unprocessed pellicle (ExSUP) pathway first, followed by the ex situ ‘suspension solution’ (ExSSuSol) pathway, to demonstrate the necessity for differentiation. 

**Figure 10:**
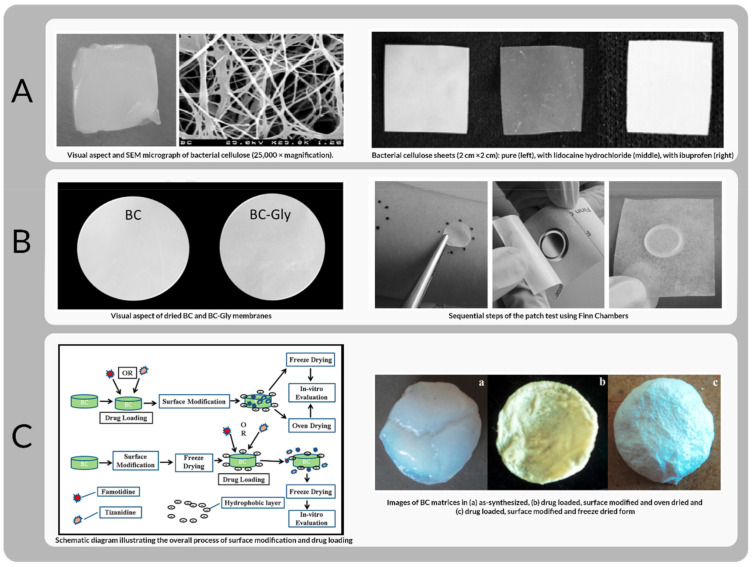
Images adapted from key studies using the ex-situ (ExSUP) modification method. (**A**) SEM micrograph of bacterial cellulose and BC sheets treated with lidocaine hydrochloride (middle) and ibuprofen (right); adapted with permission from [16]. (**B**) Visual aspect of dried BC and BC-Gly membranes and Sequential steps of the patch test using Finn Chambers; adapted with permission from [30]. (**C**) Schematic diagram illustrating the overall process of surface modification and drug loading and Images of BC matrices in (**a**) as-synthesized, (**b**) drug loaded, surface modified and oven dried and (**c**) drug loaded, surface modified and freeze-dried form; adapted with permission from [168].

Under the ex situ unprocessed pellicle (ExSUP) category, dipping, sometimes referred to as ’impregnation’ of pellicle into solvents, is one of the simplest methods for fabrication. This fundamentally requires introducing the already-derived pure BC into external molecular solvents or substances. Following that, the solvents or substances alter the molecular or physiological state of the BCs introduced into them. Irradiation and electrospraying are also techniques with high yield and reproducibility, along with various approaches discussed further below. Topical/transdermal drug delivery systems were produced by Trovatti and co. via wet BC membrane ex situ (ExSUP) impregnation with drug models ibuprofen and lidocaine [16]. The procedure involved soaking the drained BC’s in the drug solutions and agitating them to ensure full absorption. For characterization, a homogeneous-looking membrane was created (demonstrating minimal change to the BC’s morphology), which ultimately proved suitable for cutaneous applications (See Figure 10A).

BC-glycerine (BC-Gly) membrane discs were prepared by soaking BC discs in glycerine. As can be seen in Figure 10B [30], BC with glycerine provided a statistically higher skin moisturizing effect than pure BC, making it ideal for drug topical delivery to treat skin psoriasis and atopic dermatitis. Again, the drug-loaded membranes were homogeneous without the formation of drug aggregates on the surface, making them suitable for dermal applications. Ex situ (ExSUP) modification of BC to induce changes in BC membranes was done by y-irradiation with (tetracycline) as a drug model for controlled drug release [167]. BC matrices were prepared (via “ExSUP”) by using a disc fabricator, immersed either in a solution of famotidine or tizanidine as drugs [168], refer to Figure 10C. All these employed the simple “dipping” or “soaking” process.

Aris et al., engineered BC-SSD membranes through an ex-situ (ExSUP) modification method by immersing BC pellicles in various concentrations of SSD solution. The BC-SSD had pronounced antibacterial activity against *Escherichia coli* and *Pseudomonas eruginosa*, with the capability of being an alternative wound dressing for diabetic foot ulcers (DFU) [169].

A bioactive and bioabsorbable membrane was engineered with the drug model chlorhexidine (CHX) chosen. NaIO_4_ was used as an oxidizing agent. To modulate CHX release and efficacy, inclusion complexes of CHX with B-cyclodextrin (CHX:BCD) were synthesized [70]. CHX had strong chemical interaction with cellulose structure after dried BC was placed in NaIO4 solutions for oxidation before getting immersed in 15 mL of 2% chlorhexidine aqueous solution in a petri dish.

**Figure 11:**
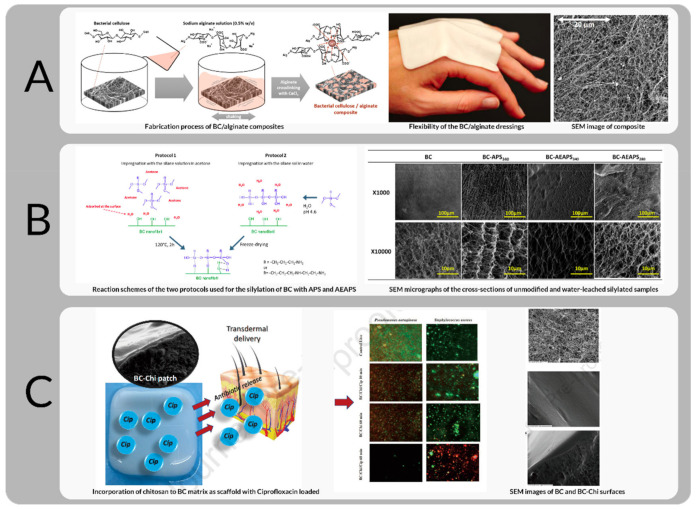
Images adapted from key studies using the ex-situ (ExSUP) modification method. (**A**) Fabrication of BC/alginate composites, image of final membrane and SEM image; adapted with permission from [172]. (**B**) Reaction schemes of the two protocols used for the silylation of BC with APS and AEAPS and SEM micrographs of composites; adapted with permission from [33]. (**C**) Chitosan-bacterial cellulose patch of ciprofloxacin for wound dressing, images observed with an epifluorescent microscope and SEM images; adapted with permission from [171].

Carbon quantum dots-titanium dioxide (CQD-TiO2) nanoparticles (NP) were added to BC as antibacterial agents [170]. Bacterial cellulose films were dipped into chitosan solution with ciprofloxacin loaded onto the BC-Chi films (see Figure 11C) for enhanced antimicrobial activity [171]; alginate was distributed evenly throughout the cross-section of the BC dressing by impregnation and showed superior stability in the substrate matrix [172], see Figure 11A.

Composites were prepared from BC films or powder and solutions of poly(3-hydroxybutyrate-co-4-hydroxybutyrate) (BC/P(3HB/4HB)). These hybrid composites were constructed using different methods [173]: Chantereau et al. report a convenient method of grafting non-leachable bioactive amine functions onto the surface of BC nanofibrils via a simple silylation treatment in water, refer to Figure 11B. Two different silylation protocols, involving different solvents and post-treatments, were envisaged and compared, using 3 aminopropyltrimethoxysilane (APS) and 2-aminoethyl)-3-aminopropyl-trimethoxysilane (AEAPS) as silylating agents [33]. BC soaked in lauric acid (LA) solutions at different concentrations [174]; BC/collagen composites prepared by immersing wet BC pellicle in collagen solution followed by a freeze-drying process [175]; amongst numerous studies, can be classified under the Ex situ ‘unprocessed pellicle’ (ExSUP) pathway

(c) Ex situ “suspension/solution” (ExSSuSol) pathway

The use of homogeneous solution or suspension of BC offers several BC-modification potentials in the biological sector. Depending on the size of the particle or fiber of choice, the BC may be synthesized as a molecular dispersion (solution), colloidal dispersion, or coarse dispersion form. In the near future, liquid dispersions are likely to be used more often in biological applications than solid-state processes [6]. Examples of aqueous dispersions found in diverse locations include the following: The reaction has been demonstrated by a clever synthesis that utilizes radiation to produce radicals, making the process practical. Freeze-drying is an excellent approach for a wide range of substances, which are dried by sublimation and condensation of the solvent molecules. Microemulsions and layer-by-layer self-assembly are other ways to go about the problem. 

**Figure 12:**
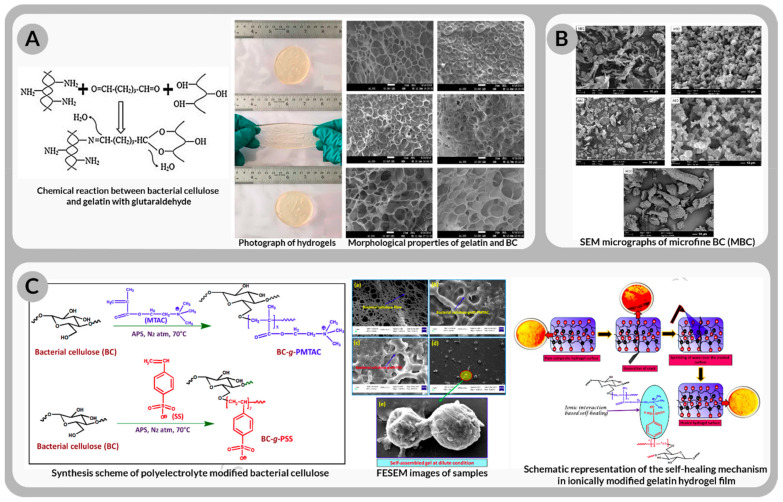
Images adapted from key studies using the ex situ “suspension/solution” (ExSSuSol) modification method. (**A**) BC/gelatin hydrogel composite formulation for drug delivery; adapted with permission from [176] (**B**) Purification, characterization and comparative studies of spray-dried BC microparticles; adapted with permission from [149] (**C**). Curcumin entrapped in gelatin/ionically modified BC-based self-healable hydrogel film adapted with permission from [177].

Ex situ “suspension/solution” (ExSSuSol) can be a time consuming and expensive method. A bacterial cellulose and gelatin-based hydrogel composite was successfully synthesized owing to the reaction between bacterial cellulose and gelatin [176]. A densely packed porous structure was developed throughout the material (clearly evidenced by Figure 12A), resulting in increased mechanical qualities and a good controlled-release capability. Figure 12B shows BC granules from nata de coco was used to make a spray-dried BC composite that has the potential to be used as a medicinal excipient. The flow rate of these BC microparticles was 4.23 g s^−1^ and they possessed a semispherical form capable for drug holding and delivery [149]. A gelatin-based hydrogel patch made of ionically modified self-assembled bacterial cellulose (iBC) derived from Gluconacetobacter xylinus (MTCC7795) bacterial strain for transdermal drug delivery is another example of an ex situ “suspension/solution” BC-composite [177]. Following the morphological experiments, microscopic sized spheres were observed, showing the composite’s ability to carry medicines (see Figure 12C).

Irradiation is the process of exposing a material to radiation. Exposure may come from a variety of sources, including natural ones. To enhance material properties, it may be used to cross-link polymers or other compounds. Homogenously ground BC was combined with different proportions of acrylic acid (AA) to fabricate hydrogels by exposure to accelerated electron-beam irradiation at different doses [178]. Electron beam processing is often employed in the irradiation treatment of polymer-based materials due to its effectiveness in improving mechanical, thermal, and chemical characteristics, as well as adding unique features. Lyophilized BC was ground to a powder of particle size between 20 and 200 um before getting irradiated. The water molecules were transformed into reactive species during the irradiation process, such as electrons, radicals of hydroxyls, and hydrogen atoms, which produced active AA and BC grafting sites. According to morphological studies, the extremely porous sponge-like structure of the BC/AA hydrogels promoted water diffusion in all directions, making the hydrogels ideal for drug administration.

Pickering emulsion method was used by Yan and co. for interfacial assembly of amphiphilic bacterial cellulose to improve the compatibility between the alginate and hydrophobic drug. The resultant alginate composite beads exhibited low cytotoxicity and good capabilities for osteoblast differentiation [37]. The BC suspension in this experiment was hydrolyzed and later oxidized before the emulsion formation was done with simple chloro-hydrocarbons, CH_2_Cl_2_, and alfacalcidol. The derived drug-loaded Pickering emulsion was finally dispersed in alginate solution to complete the procedure. The composite beads performed well in terms of sustained release.

Bacterial cellulose-graft-poly(2-(methacryloyloxy)ethyltrimethyl ammonium chloride) (BC-g-PMTAC) was derived; initially bacterial cellulose (1 wt %) was dispersed in alkaline distilled water solution, mechanically stirred to a homogenous dispersion.

(d) Hybrid pathway

**Figure 13:**
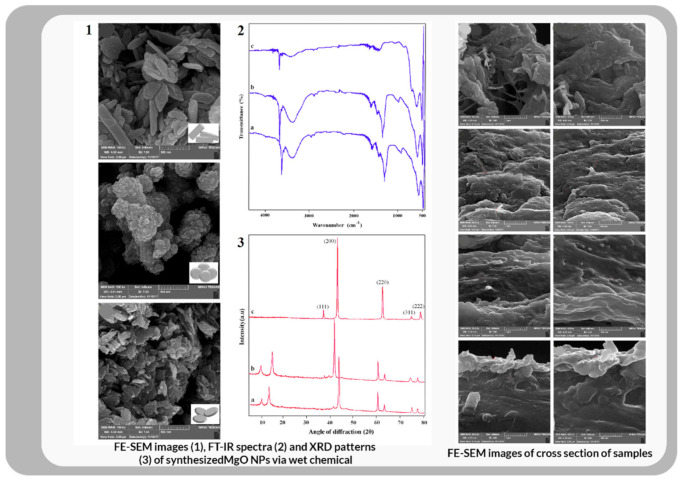
Image of novel MgO-BC nanohybrids prepared by in-situ and ex-situ methods using the hybrid modification method; Adapted with permission from [147].

Mirtalebi and colleagues have conducted a fairly revolutionary experiment. MgO-bacterial cellulose (BC) nanohybrids were fabricated by both in situ and ex situ synthesis of nanoparticles (NPs) within the BC network. The ex situ synthesis was prepared by immersing BC pellicles in a commercial MgO dispersion. Inside the BC network, nanoparticles (NPs) were synthesized in situ using two methods: sonochemical and wet chemical [147]. The crystalline structure of BC was maintained after MgO impregnation through the ex-situ and wet chemical in situ methods, but the crystallinity parameters of BC were significantly changed by the sonochemical in-situ process (see Figure 13). According to SEM data, the MgO-NPs entered the inner spaces of the BC matrix using in situ processes but agglomerated on the surface of the ex-situ synthesized nanohybrid. The structural properties of the nanohybrid showed that it has potential applications in a wide range of industries, including biofilms, food processing and packaging, water treatment, and, most importantly, drug carrier systems for therapeutic wound healing.

#### 2.3.3. Some Modes of BC Modifications for Drug Delivery 

##### Modification via Cross-Linking Reactions

Cross-linking has proven over time to be an effective method for the improvement of BC with desirable properties. Cross-linkers are appropriate for biopolymer materials, particularly those obtained from proteins or carbohydrates. They have also been found to supply reduced gas and water vapor permeability in food packaging materials [179]. Through this mechanism, many polymers, either naturally or synthetically, are modified to experience an increase in their potential range of applications. Polymer chains get interconnected by covalent or non-covalent links, helping make up for the intrinsic deficiencies in the barrier and mechanical properties of biopolymers, rendering them more applicable in comparison with their petroleum-based counterparts [180]. Liang and co. report that, generally, improved mechanical properties, heat stability, and water resistance are obtained by cross-linking, whilst the qualities of composites can be controlled by means of adjusting the mode or extent of cross-linking [181]. Many BC composites have been successfully utilized for various functions after they underwent such reactions [182,183,184], for example, getting cross-linked with fibrin in the presence of glutaraldehyde. The cross-link was confirmed to have been formed between the hydroxyl groups and amine groups found on BC and fibrin, respectively [185].

##### Modification via Grafting

Chemicals generally possessing protonated nitrogen (N+) are often used as antibacterial agents to be grafted onto the BC; they include amine, quaternary ammonium, and amino. The N+ is said to neutralize the negative charges from the phospholipid bilayer in the cell membrane, destroying the integrity of the cell membrane and offering antimicrobial capabilities to biomaterial surfaces [108]. This is a grafting process. Grafting onto or from BC extends the potential for surface modification. Acrylic acid was grafted onto BC with the use of ionizing electron beam radiation for potential oral drug delivery in a controlled manner at intestinal pH [178], whereas in another report, acrylamide was grafted onto BC using microwave radiation [186]. Grafting effectively enhances the positive surface charges of some cellulose polymers to help them acquire excellent antimicrobial efficacy.

##### Modification via Mineralization on and across the Fiber

Coelho and colleagues created a ground-breaking BC membrane with hydroxyapatite (HA) and an anti-bone morphogenetic protein antibody (anti-BMP-2) (BC-HA-anti-BMP-2) with fascinating physical-chemical and biological properties for bone regeneration to facilitate improved bioactivity against BC [187]. Several experiments have been conducted in order to create a clear bond between engineered material and natural bone tissue. This involves the biomimetic mineralization of a hydroxyapatite layer. Polymers with hydrophilic polar (e.g., hydroxyl, carboxyl, and silanol) groups are used due to their capacity to induce apatite nucleation in bone regeneration therapies [188]. A cellulose bone biomaterial alternative was developed by first applying PVP (polyvinylpyrrolidone) treatment, followed by biomimetic mineralization. The engineered hydroxyapatite HAp/BC composite, as opposed to genuine bone apatite, bore semblance to the natural alternative by way of their intrinsic physical characteristics. They are an excellent bone biomaterial replacement since they have the potential to eventually develop these qualities after nucleation [189]. Another study done by Tolmachev and his colleagues shows that bacterial cellulose can be mineralized using a mixture of CaCl_2_ solutions. The results of this experiment generated the nucleation of BC fibrils to produce crystallites for future biomimetic systems. This family of innovative bio-based materials made from bacterial cellulose and calcium phosphates can be great alternatives for tissue engineering and surgical procedures due to their outstanding tensile strength, robust osteoconductivity, and biodegradability [190].

##### Reactivity via Hydroxyl Sites

According to Abeer et al., the primary hydroxyl group of cellulose is not very reactive to hydroxyapatite, so surface alteration by phosphorylation can be very useful in biomedical applications [20]. For alteration of BC microfibrils, the main hydroxyl group can be oxidized to a carboxyl group, as seen in 2,2,6,6-tetramethylpiperidine-1-oxyl (TEMPO)-mediated oxidation [191]. BC was also prepared as an ionic solvent as a homogeneous solution with chemical reactions involving all three hydroxyl groups, resulting in the alteration of BC by acetates and carbanilates [192]. Many further therapeutic uses of BC can be aided by hydroxyl-site reactivity. With grafted copolymers of poly(lactic acid) (PLA), organic acids, glycidyl methacrylate, and other materials, BC has been given hydrophobicity, modulated contact angle, and improved mechanical properties [193]. 

##### Modification via Etherification and Esterification

Furthermore, BC can interact with various molecules facilitated by etherification and esterification to introduce further advantageous properties [194,195]. Suspensions of BC in organic solvents with amine functionalization had an added benefit, as stated with the use of hexamethylene diisocyanates to cause hydrophobicity on BC [196]. Mustafa and colleagues show that esterification of BC necessitates the interaction of a special activated carboxylic acid salt and that BC must be dissolving to undergo esterification [20]. It has been reported that acetylated BC was esterified with poly(lactic acid) PLA to generate a nanocomposite with attributes such as enhanced tensile toughness and good resistance to UV degradation, which are similar to those of many natural polymers. Furthermore, a glossy finish achieved established the BC/PLA composites’ potential usage as drug holders and delivery units [197]. Research in the past several years has focused on how to impart hydrophobicity to cellulose microfibrils with their hydroxyl groups replaced by less hydrophilic ester groups. Due to the surface hydroxyl groups of microfibrillated cellulose (MFC), it was possible to perform a monolayer reaction of the respective anhydrides with the associated succinic and maleic acid groups [196]. For delivery systems to be used in the intestinal system, a polyelectrolyte complex made up of chitosan and sodium cellulose sulfate (NaCS) was engineered with their biodegradability explored using the enzymes trypsin, cellulase, amylase, pepsin and lipase. They proved to be ready substitutes for oral administration of drug models based on their degradation performance [198].

Four different forms of alkyl-BC (n-butyl, ethyl, propyl, isopropyl) were synthesized in the presence of a lithium chloride/dimethyl acetamide solution, followed by etherification of the resulting mixture [195]. 

## 3. Perspectives, Challenges and Future Prospects for BC

BC has reached as far as the FDA approving it as a component of wound dressers and other products [20]. Becoming a commercial consumable for that matter is a testament to how far it has come over the years. Evidently, BC could be used for drug delivery systems, tissue-engineered scaffolds, other transdermal applications, and even as pharmaceutical excipients in many areas of biomedicine. Undeniably, BC and its composites have found multi-disciplinary usage at such a respectable level. However, its continued acceptance and industrial level utilization begs the question, “Can BC replace, or at least, beat its well-established competitors like collagen and the conventional cellulose variants for drug delivery and more?” The FDA’s approval of BC for wound dressing instils ever-growing confidence in researchers around the globe to extend its exploration for usage in the other drug delivery categories. Throughout this paper, we have discussed many great drug carriers and ground-breaking successful utilizations of BC and its membrane composites for biomedical applications. The unique properties of BC served as the reason for the successes, as well as the intuitive modifications from the researchers with other molecules and materials, which further augmented the primal characteristics of BC and have opened even more possibilities for its usage in many delicate medical sectors. This makes the exploration of BC more exciting in our current era. 

Yet, there are still vital hurdles to surmount in the laboratories and industries to make BC and its composites unconditional go-to materials for drug delivery and biomedical products. First of all, the scaling up of BC for commercial use needs to be more advanced; the same goes for its fermentation bioprocesses. The emergence of bioreactors for industrial scale production of BC was a great breakthrough a few years ago, but they are still not economically feasible [122]. Sharma and co. have done a great job of breaking down the issues with BC into four main categories. They are: (i) production-based challenges, (ii) Substrate-based challenges, (iii) strain-based challenges, and (iv) clinical progress and marketing challenges. In our many years of research on BC and prior research to write this review, we undisputedly agree with these points identified by Sharma and co. From scores of papers read on BC for drug delivery and biomedical applications, it is very obvious that the tackling of the issues should begin at the production stage. Researchers have discovered that, while *Gluconacetobacter xylinus* strains appear to be the most commonly used species (due to their high production capacity and speed of production), other strains have proven to be very capable if cultivated or engineered under optimal conditions and with rich nutrient sources such as carbon sources and other valuable supplements. The production level challenge lies with scaling the production quantity, where bioreactors seem to have become the solution. However, they are very expensive. Also, there seems to be a need for further studies on bioreactors to optimize production and improve their designs for proper control of the pH and temperature. Sharma and co. [8] suggest that if the pH, oxygen and temperature can be maintained at optimum values throughout the fermentation, the production of microbial cellulose might attain an increment. Due to the expensive nature of bioreactors, explorers have stuck with the more basic modes of production, which is either the static mode or the agitated mode. For this reason, researchers have not taken full control of the production of BC and its composites, which still calls for more intense studies. 

The substrate-based challenges have to do with first seeking less expensive composites that can be highly performing in the production of the cellulose from the bacteria. Many researchers are turning to agricultural by-products and alternatives, which are helping to reduce the cost of substrates impressively. Production speed is as essential as the production quantity or capacity. On average, it takes between 3 and 7 days to harvest a desirable quantity or thickness of the BC pellicles. In the economy of scientific research, reducing this time drastically will represent a great feat. Which means, further exploration is highly recommended. The use of cheap agricultural by-products is proving to be environmentally friendly as well, which is globally welcomed above anything else. 

As seen throughout this review, the strain or genera of bacteria is a very key contributing factor in getting a maximum quantity of cellulose within a short space of time, with desirable properties for the end-application of the BC material. As identified earlier, the *Gluconacetobacter xylinus* group has been the most used and successful genus, even more so when they are co-cultured with less expensive by products from industries. More and more new species of bacteria are being experimented on to see how they can beat the acetobacter specie in efficiency and productivity. A more productive and robust species will be a game changer if they can be cultivated to be hugely productive within a day of cultivation (producing more than 50g/L) and still demonstrate great chemical and physical properties, as well as be capable of producing cellulose during fermentation process. 

Furthermore, under strain-based efforts, greater efforts are needed to understand the interactions among different microbial groups, their combined effect on the production of BC, their physico-chemical properties, shelf stability, how they affect production yield, and the molecular mechanism of polymerization of glucose into long unbranched chains. Not only that, the supramolecular structure of the catalytic and regulatory protein complexes involved in the BC synthesis still requires further exposition through rigorous studies.

The clinical studies and marketing-based challenges of BC and its composites entail researchers understanding and exhaustively interpreting the results of in vitro assays and animal studies into clinical applications for humans. It is important to state here that this is the main bottleneck in the way of introducing BC composite DDS and biodevices to the market. Sharma and co. reveal that a large number of BC-based therapeutics are available but many of them are not yet approved for patients’ benefit [122]. They further state that the major reasons for the small numbers of approved products in the mainstream are issues with the scaling-up at the manufacturing level, the cost of development, sterility issues and patent apprehension. Furthermore, challenges in the area of clinical deployment and marketing have to do with the fact that many regulatory barriers exist along the way, like quality assertion for consistent manufacturing, quality control, and comparability evaluation needed for component and process changes. A more marketing-related issue involves establishing shipping and storage conditions for the new products as well as shelf-life appropriation. 

We add here that, at the laboratory level, exploration of BC, especially BC composites for DDS and other biomedical usage, overcoming biodegradation issues, establishing greater control on the porosity, maintaining quality consistency of cellulose producing bacteria, and ensuring structural diversity between the outer surface layer and internal parts to aid in drug holding and controlled release needs advancement in exploration.

## 4. Conclusions

This paper reviewed the latest developments made in the drug delivery categories of biomedicine using BC. Bacterial cellulose has proven to be a promising natural polymer with many biomedical applications, especially for drug delivery in recent years. This work reported on membrane technologies, nanomembranes for biomedical applications and DDS routes and technologies, then expressly reviewed bacterial cellulose membranes, structures, patents, and commercial BC-DDS, and then the critical aspects that are vital for BC-DDS and biomedical applications. The strains of bacteria, biosynthesis pathways, the intrinsic properties of BC-DDS composites, successful applications, and frontier research on BC have all been discussed in detail.

In a conclusive manner, it could be seen from Table 5 that most studies have involved in situ and ex situ pathways in the fabrication of BC-DDS. A hybrid approach is slowly being explored. Under the ex situ method, the unprocessed pellicle approach has found more utilization than the solution/suspension approach which involve an extra effort to process the harvested BC membranes to granules or chips or powder to make suspensions or solutions. Ex situ (ExSUP) is straightforward and cost-effective. The morphological features of the systems were insightful. Most BC-DDS intrinsic features, according to the publications we reviewed, revealed that the drug models were mostly incorporated into the 3D nano-fibrils of BC without drastically changing the morphology. However, most characterizations showed fleece-like features, bead-like spheres, pockets/nano-spheres for encapsulation of drugs, leaf-shaped nano-sheets and sponge-like appearances. Deep within the structures, the drug hold and release were discovered to be facilitated by hydrogen bond interactions with hydroxyl groups of BC, ionic interactions, the availability of hydrophobic/hydrophilic backbones on BC components and the modifiers and or drug models, the formation of mid-chain radicals at crosslinked sites, etc.

Furthermore, the predominant drug administration routes with BC-DDS were mostly transdermal, transmucosal, and oral channels, although there were few reports on delivery by topical routes. Transdermal appears to be the best route of administration as far as BC is considered. 

As was apparent earlier on, *Gluconacetobacter xylinus* is the most commonly used strain of bacteria for most drug-carrying systems. BC DDS has been most successful for wound dressings and tissue regeneration, but has been adequately successful as drug capsule film, dental scaffolds and other therapeutic drug carriers.

This indicates that BC could be applied to more areas than was previously thought, and a multidisciplinary approach is required to fully exploit the drug delivery potential of BC.

## Figures and Tables

**Table 1:** Commercial bacterial cellulose products available on the market [11,20].

Commercial Product Name	Clinical Utilization	Form for Usage	Company/Agency
Bio Fill^®^	Burns	Wound care systems	Robin goad, Milwaukee, WI, USA
Cellulon^®^	Medical applications including non-woven structures	Binder	CP Kelco, Atlanta, GA, USA
Basyc^®^	CABG (Coronary artery bypass surgery)	Vessel implants (tubes)	Jenpolymer materials Ltd. & co., Jena, Germany
Bioprocess^®^	Burns	Artificial skin	Biofill Produtos Biotechnologicos, São Paulo, Brazil
Dermafill^®^	Burns	Wound care dressing	Fibrocel Produtos Biotechnologicos Ltd.a, Ibipora, PR, Brazil
Cellulon PX microfibrous cellulose^®^	Suspensions of particles, encapsulated enzymes	Suspending agent	CP Kelco, Atlanta, GA, USA
Gengiflex^®^	Periodentitis	Non-resorbable cellulose membrane	Biofill Produtos Biotechnologicos, São Paulo, Brazil
CelMat ^®^ MG & CelM^®^(R) MG	Protection for miners from potential burns	Protective dressings/jackets	Government of Poland, Warsaw, Poland
Securian^®^	Tendon repair	Tissue reinforcement matrix	Xylos corporation, Langhorne, PA, USA
MTA protective tissue	Injury and wound care	Biocompatible implant	Xylos corporation, Langhorne, PA, USA
Membracell^®^	Ulcers, burns, lacerations	Temporary skin substitute	Vuelo Pharma, Curitiba, PR, Brazil
Xcell^®^	Venous ulcer wounds	Wound care	XCELL BIOLOGIX, Kennesaw, GA, USA
Bionext^®^	Ulcers, burns, lacerations	Wound dressing	Bionext Produtos Biotechnologicos, Pacaembu, São Paulo, Brazil

**Table 2:** Drug delivery patents on BC [20].

Invention Field	Patent Title	Patent Number	Registration
Calcium alginate capsules embedded and prepared in situ; containing drugs and probiotics	Bacterial cellulose composite with capsules embedded therein and preparation thereof	US 2012308649A1	United states patent and trade mark office (USPTO)
Implantable device; soft tissue repair-drug delivery carriers	A method for producing implantable microbial cellulose materials for various medical applications	EP1795213 B1 (Heather Beam et al.)	European patent office
Network meshed hydrogel, drug delivery carrier, skin substitute	Novel network meshed hydrogel structure	TW M428771U1 (Yung Kai Lin, Che Yung Kuan)	Intellectual Property Office Taiwan (TIPO)
Implantable bacterial cellulose; in-vivo application	Thermally modified microbial-derived cellulose for in-vivo implantation	EP1662976 A2 US20050042250 US8198261, (Ann Hethearbeam et al.)	USPTO, 2006 & EPO, 2005
Use of microbial (bacterial) cellulose in transdermal drug delivery	Microbial cellulose materials for use in transdermal drug delivery systems, method of manufacture and use	US 20060240084 (Serafica et al.)	USPTO, 2006
Cellulose hydrogels, making and applications; implant and ocular devices; sustained release drug delivery systems	Cellulose-based hydrogels and methods of making thereof	US20130032059 A1 (Morgana M Trexeler et al.)	USPTO 2013
Medical implant; orthopeaedic	Medical device including bacterial cellulose reinforced by resorbable or non-resorbable materials	US 20110262521A1 (Bayon et al.)	USPTO, 2011
Wide range of applications, dependent on density gradient dictated by thickness; number of drugs can be delivered	Bacterial cellulose films and uses thereof	EP 2390344 A1 US20110286948 (Mei-Ling Lee et al.)	EPO, 2011 USPTO, 2011

**Table 3:** Cellulose-producing bacteria strains that have been heavily studied [84,87,122].

Strain	Carbon Source	Production Quantity (g/L)	Incubation Mode	Duration of Incubation	Reference
G. *xylinus (BPR 2001)*	Fructose	14.1	Agitated	3 days	[123]
G. *xylinus (BRC 5)*	Glucose	15.3	Fed-batch/agitated	2 days	[124]
G. *xylinus (MCRC 12334)*	TS-Glu	10.38	Static	7 days	[125]
A. *xylinum (ATCC 700178)*	CSL-Fru	13	Agitated	5 days	[126]
*G. xylinus (ATCC, 23770)*	(Fiber sludge) Hydrolysates	6.23	Static	14 days	[127]
*G. xylinus (PTCC 1734)*	Syrup	43.5	Static	14 days	[128]
*Acetobacter xylinum ssp. sucrofermentans BPR2001*	Fructose	8.7	Static	44h	[129]
*Gluconacetobacter xylinus IFO 13773*	Glucose	10.1	Static/agitated	7 days	[130]
*Acetobacter sp. V6*	Glucose	4.16	agitated	8 days	[131]
*Acetobacter sp. A9*	Glucose	15.2	agitated	8 days	[132]
*Gluconacetobacter xylinus IFO 13773*	Sugar cane molasses	5.76	Static/agitated	7 days	[133]
Co-culture of *Gluconacetobacter sp. st-60–12*	Sucrose	4.2	agitated	3days	[134]
and *Lactobacillus mali JCM1116*					
*G. hansenii PJK (KCTC 10505 BP)*	Glucose	2.5	Static	3days	[76]
*A. xylinum 0416*	Pineapple waste medium	28.3	Rotary disc reactor	4 days	[135]
*A. xylinum strain DA*	Glucose	0.15	Five-stage horizontal	68 h	K Toda, J Koizumi, T Asakura—1994
			flow reactor		
*A. xylinum subsp. Sucrofermentans BPR2001*	Corn steep liquor-fructose (CSL-Fru)	3.8	Airlift reactor	67h	[129]
	medium				
*G. persimmonis GH-2*	Galactose + Sucrose	7.67	Static	14 days	[136]
	Galactose + Lactose,	6.89			
	Galactose + Maltose,	6.28			
	Galactose + Fructose	5.82			
	Molasses + HS medium	5.75			
	Watermelon + HS medium	5.98			
	Orange juice + HS medium	6.18			
	Muskmelon + HS medium	8.08			
	Coconut water + HS medium				

**Table 4:** BC Cultivation approaches. Adapted with permission from [122].

Production Method	Description	Advantage	Disadvantage	
Static culture	-All media ingredients are mixed together at the early stage	-Simple process	-Laborious and time consuming	All references can be found in [122]
	-Production occurs in tray	-Does not require complex instruments	-Fermentation condition cannot be controlled or monitored	
	-Production occurs at air-liquid medium interface		-Cellulose formed as pellicle, sometimes as reticulated cellulose	
			slurry	
			-Not applicable for large-scale production	
Static intermittent fed batch technology	Definite amount of fresh media provided over growing	Simple process	-Fermentation condition cannot be monitored	
	pellicle in intermittent time periods	-Highly enhanced production as compared to	-Cellulose formed as pellicle, sometimes as reticulated cellulose	
		standard static method	slurry	
		-Can be applied for large scale production		
Cell-free extract technology	Mechanical/thermal/enzymatic cell lysis releases all the	Simple process	No control over fermentation parameters	
	necessary enzymes required for BNC production directly	-Can be applied for large scale production in		
	into the media	short time		
		-Better yield		
Agitated culture	-Reciprocal shaking at about 90–100 rpm	-Applicable for large scale production	-Cellulose not formed in pellicle form but as irregular shape	
	-Agitation allows cells to grow more rapidly	-Surmount many limitations in static culture	sphere-like cellulose particle	
		including diffusion, controllability and scale-up	-Agitation often result in culture mutation resulting in low	
			productivity	
			-Problem with culture instability which demonstrated by loss of	
			ability to make cellulose	
Bioreactor based production e.g., Rotary disc	New alternative using concept of Rotating Biological	-High productivity		
reactor, Air lift reactor	Contactor (RBC)	-Less labor needed	-No disadvantage (if culture conditions are properly maintained	
	-It used discs that alternately soak the organisms in nutrient	-Easy scale-up	and suitable medium is used then high productivity can be	
	medium and expose them to air		achieved)	

**Table 5:** Preparation methods and strategies for BC DDS membranes.

Mode of Modification	BC Strain and Drug Model	Intrinsic Feature	Final Application	DD Route	Reference
In situ	*Komagataeibacter xylinus (K. xylinus) strain DSM 14666* (doxycycline)	Fleece-like appearance	Wound dressing and dental therapies	Transmucosal delivery	[158]
In situ	*Komagataeibacter xylinus X-2* (graphene oxide)	Bead-like spheres with BC/GO porous structure	General carrier	Potentially for transdermal and transmucosal drug delivery	[159]
In situ	*Gluconacetobacter xylinus (ATCC 10,245)*	Pockets	Drug carrier	For transdermal and transmucosal drug delivery	[138]
In situ	*Acetobacter xylinum (ŁOCK 0805)*	3D microfibres	Dressers for wounds, burns and ulcers	Transdermal	[161]
In situ	*Gluconacetobacter xylinus* (hydroxyapatite Ca5(PO4)3OH (HA)	Nanotextured fibrils	Varied applications	Mainly transdermal	[162]
In situ	*Gluconacetobacter xylinus*(magnetite nanoparticles (Fe3O4))	Nanotextured fibrils	Blood vessels	Potentially for transdermal and transmucosal drug delivery	[163]
Ex situ (ExSUP)	*Gluconacetobacter sacchari* (ibuprofen and lidocaine)	3D microfibrils	Drug carrier absorb exudates skin therapies	Transdermal	[16]
Ex situ (ExSUP)	*Gluconacetobacter sacchari* (glycerine)	3D microfibrils	Skin therapy	Transdermal	[30]
Ex situ (ExSUP)	*Acetobacter Xylinum* (tetracycline diffusion) via irradiation	3D microfibrils	Varied applications	Potentially for transdermal delivery	[167]
Ex situ (ExSUP)	*Gluconacetobacter xylinus (ATCC No. 23769)*(digluconate chlorhexidine)	3D microfibrils	Varied applications	Potentially for transdermal delivery	[70]
Ex situ (ExSUP)	*Acetobacter Xylinum 0416* (silver sulfadiazine)	Nano-spheres with 3D microfibrils of BC	Wound dressing for diabetic foot ulcer (DFU)	Transdermal delivery	[169]
Ex situ (ExSUP)	*Komagataeibacter hansenii* (2,3-dialdehyde + chlorhexidine)	Nano cavities with BC microfibrils	Bioabsorbable membrane/periodontal treatment	Potentially for transdermal and transmucosal drug delivery	[70]
Ex situ (ExSUP)	*Gluconacetobacter xylinus (PTCC 1734)*(carbon quantum dots-titanium dioxide (CQD-TiO2)	3D microfibrils	Wound healing	Transdermal delivery	[170]
Ex situ (ExSUP)	*Komagataeibacter xylinus (ATCC 23760)*(Chitosan) (Ciprofloxacin)	3D microfibrils	Wound treatments	Transdermal delivery	[171]
Ex situ (ExSUP)	*Gluconacetobacter xylinus* (alginate)	3D microfibrils	Wound dressing	Transdermal delivery	[172]
Ex situ (ExSUP)	*Komagataeibacter xylinus B-12068* P(3HB/4HB)	Nanotextured fibrils	Wound treatments	Transdermal delivery	[173]
Ex situ (ExSUP)	*Gluconacetobacter sacchari* (Silylation)	3D nanotextured fibrils	Anti-bacterial activity	Transdermal delivery	[33]
Ex situ (ExSSuSol)	*Acetobacter xylinum* (Gelatin)	Spherical porous structure	Drug carriers	Transdermal and transmucosal drug delivery	[176]
Ex situ (ExSSuSol)	*Acetobacter xylinum* *(CGMCC5173)* (alfacalcidol via pickering emulsion method)	Spherical (bead-like) nanocrystals	Drug carriers	Transdermal and transmucosal drug delivery	[37]
Ex situ (ExSSuSol)	*Acetobacter xylinum* (Acrylic acid (AA))	Sponge-like structure	Drug carriers	Potentially for transdermal and transmucosal drug delivery	[178]
Ex situ (ExSSuSol)	*Glucanoacetobacter xylinus (MTCC7795)* (cellulose-graft-poly(2-(methacryloyloxy)ethyltrimethyl ammonium chloride) (BC-g-PMTAC))	Spherical (bead-like) nanocrystals	Drug carriers	Transdermal and transmucosal drug delivery	[177]
Hybrid pathway (In situ+Ex situ)	*Gluconacetobacter xylinus* (MgO)	Leaf-shaped nano-sheet structure	Clinical wound healing	Transdermal delivery	[147]
